# Stem cell ‘therapy’ advertisements in China: Infodemic, regulations and recommendations

**DOI:** 10.1111/cpr.12937

**Published:** 2020-11-04

**Authors:** Jianwei Lv, Yeyang Su, Lingqiao Song, Xia Gong, Yaojin Peng

**Affiliations:** ^1^ State Key Laboratory of Stem Cell and Reproductive Biology Institute of Zoology Chinese Academy of Sciences Beijing China; ^2^ Institute for Stem Cell and Regeneration Chinese Academy of Sciences Beijing China; ^3^ Center of Genomics and Policy McGill University Montreal QC Canada; ^4^ University of the Chinese Academy of Sciences Beijing China

**Keywords:** stem cell, advertisement, infodemic, regulation, China

## Abstract

During the COVID‐19 pandemic, in addition to the pandemic itself, a phenomenon called an ‘infodemic’—defined by the World Health Organization as the spread of misleading information on the pandemic—has also gained attention. In the field of stem cell research, researchers and regulators have been fighting against false and misleading information, particularly advertisements for unproven and unauthorized stem cell–based interventions for decades. However, how existing legal and regulatory measures, which vary by country, can be employed to combat such false information is unclear. In this article, we examine the situation in China, where the spread of unauthorized stem cell ‘therapies’ has drawn patients from not only within China but also from abroad. First, we assess how and to what extent online advertisements promote unproven and unauthorized stem cell–based interventions directly to patients and prospective health consumers in China. Next, we survey the landscape for existing regulatory and administrative measures that may be used to combat false and misleading advertisements in this area. Finally, based on our analysis, we provide three main recommendations that may improve the effectiveness and efficiency of the regulatory measures in curtailing illegitimate advertising of unproven and unauthorized stem cell–based interventions in China. In conclusion, we also call for international collaboration among researchers and regulators in studying and strengthening regulations in this critical area that has so far been neglected in scholarly and policy discussions.

## 1 INTRODUCTION

As early as February 2020, the World Health Organization warned about the spread of false and misleading information and the adverse impact an ‘infodemic’^1^ might have on efforts to combat the COVID‐19 pandemic.^2^ While scientists, health professionals and pharmaceutical companies have been ceaselessly working to develop viable diagnostic methods, treatments and vaccines, advertisements for unproven and unapproved products have appeared on the Internet, beginning shortly after the onset of the pandemic.^3^ Among the various advertised treatments for COVID‐19, stem cell–based interventions are of particular concern,^4^ partly because scientists and regulators have been fighting against Internet‐based, direct‐to‐consumer advertisements (DTCA) for unproven and unauthorized stem cell–based interventions for approximately two decades.^5^

Internet‐based, direct‐to‐consumer advertisement is a key component of the business models of clinics and biotech companies selling unproven and unauthorized stem cell–based interventions, with the aim to attract patients residing domestically and abroad.^6^ These treatments pose serious safety and financial risks to patients and their families.^7^ By raising their expectations and spreading misinformation, the companies also undercut efforts to develop safe and effective stem cell–based treatments for patients whose diseases or conditions currently have no effective treatment.^8^

Although the detrimental effects of false and misleading advertising of unproven and unauthorized stem cell–based interventions have been widely acknowledged, few studies have addressed the regulatory issues related to such advertising activities in China. In this article, we take China as an example and assess how challenging it may be to combat false and misleading advertisements of stem cell–based interventions. Our findings reveal that China has relevant regulations and policy documents that can be useful in combating false and misleading advertisements in this area. Nevertheless, to successfully curtail this phenomenon, the legal status of stem cell–based interventions needs to be clarified, and relevant regulatory agencies need to work together to enforce these measures. Overseeing the development, market authorization and post‐market regulation of stem cell–based interventions is a shared challenge for regulators around the world; therefore, we consider our study the first step in systematically reviewing and comparing nation‐level regulatory measures in this area. Such joint efforts will, we hope, contribute to promoting the sustainable development and healthy flourishing of stem cell–based treatments and relevant industries in China and other countries.

## 2 ADVERTISING AND PROMOTION OF STEM CELL–BASED INTERVENTIONS IN CHINA

No stem cell–based intervention has received market authorization in China, and advertisements for such products remain illegal. Nevertheless, we used ‘stem cell therapy’ as the search term in China's largest search engine Baidu (https://www.baidu.com) and retrieved 38.9 million results (search conducted on 10 September 2020). Upon analysing the content of the first 10 pages of those search results, we identified more than half of the results as DTCA. We then analysed the content of those advertising materials. We deployed the same methods to search and analyse the advertising content on the biggest e‐commerce platform in China, Alibaba (https://www.1688.com). We identified 2596 online stores selling stem cell–related products directly to consumers and analysed their advertisements (search conducted on 14 September 2020).

The aforementioned advertising materials were generated by a range of actors, including companies, research sponsors, manufacturers, importers, pharmacists, health professionals and health services marketers. Based on the primary resources that the advertisers utilized to create their advertisements, we categorized the advertisements into three groups: those relying on case reports and patient testimonies, those highlighting the credentials of the actors and those utilizing educational materials concerning stem cell research.

First, some providers claim that unproven stem cell–based interventions can cure several serious diseases. For example, a company that mainly provides services such as cellular immunotherapy and stem cell therapy for treating diabetes and other diseases advertised on its website that it had successfully treated more than 1200 patients through a stem cell–based intervention. The company's homepage even displays a ‘guarantee of effectiveness’ and other attractive slogans.^9^ Another company claimed on its website that stem cell ‘therapy’ can effectively treat diseases and offered anti‐ageing, male and female reproductive and sexual function treatments and treatments for diabetes, osteoarthritis, gout, stroke, cardiovascular disease and cancer.^10^ Notably, the company offers two stem cell–based products for treating azoospermia and premature ovarian failure. The company also publishes patient treatment testimonies and successful cases of testicular stem cell transplantation for mumps and azoospermia on its website.

Second, some providers portray themselves on their websites as trustworthy providers of stem cell–based interventions by publicizing their ‘achievements and honours’ using various materials such as scientific publications, patents, registered clinical trials, conferences organized and certificates of various types. For instance, one company promotes that its independent research and development of ‘GLP‐1 and FGF21‐modified autologous adipose stem cells for treating type 2 diabetes’ technology has achieved a major breakthrough. It also alleged to have applied for China's National Key Research and Development Project on ‘stem cell and translation research’ and conducted clinical research.^11^ Such information is misleading. Few outside the scientific and medical fields would know that, for instance, merely registering a study on clinicaltrials.gov does not mean this study has been approved by the regulatory body in China, and thus, such registration does not constitute a legitimate clinical trial. Nevertheless, advertisers often indicate otherwise.^12^

Third, some providers use educational materials to publicize the efficacy of their stem cell–based intervention(s). Because the rigour of such educational materials varies and so‐called ‘scientific popularization’ articles often describe stem cells as a panacea, these materials are easily repurposed by providers of unauthorized stem cell therapies as evidence of the effectiveness of their products, thus misleading the public. For instance, a company that sells stem cell facials claims that its facials ‘activate the renewal of stem cells in tissues and improve skin circulation’.^13^

In all three groups, those advertising unproven and unauthorized stem cell–based interventions online take advantage of the information asymmetry between the generator and the targeted receptor of the content—in this context, the receptors are often vulnerable patients and their families. Although the considerable profit that selling such unauthorized products brings to the advertiser explains their motivation, the current regulatory situation may contribute to the scope and scale of this problem. That is the question we investigate next.

## 3 REGULATORY FRAMEWORK IN CHINA

To a certain extent, the proliferation of deceptive advertising and the promotion of unproven stem cell–based interventions have a close relationship with the current regulatory framework and enforcement in China. Thus, it is necessary to understand the relevant regulatory system before analysing its implementation or providing suggestions.

### 3.1 Regulatory scheme governing stem cell–based interventions

In China, policies and regulations governing stem cell–based interventions have reflected indecision as to whether the law considers them to be a drug or a medical technology.^14^ Under two principle regulatory documents, the Guidelines for Quality Control and Preclinical Studies of Stem Cell Preparations (Trial)^15^ and Administrative Measures for Stem Cell Clinical Research (AMSCCR),^16^ jointly issued by China's National Health and Family Planning Commission (NHFPC, formerly known as the Ministry of Health [MOH], which later became the National Health Commission [NHC]) and China Food and Drug Administration (CFDA, formerly known as the State Food and Drug Administration [SFDA], which later became the National Medical Products Administration [NMPA]) in August 2015, stem cell–based interventions are regulated as a drug in China. However, no legal opinion or court decision in China clearly defines it as a drug. Therefore, deciding whether stem cell–based interventions are a drug or medical technology critically affects the regulation of advertising and promotion in this field because they may advertise and promote stem cell–based interventions as drugs or medical services.

### 3.2 Regulatory system for the advertisement and promotion of stem cell–based interventions

#### 3.2.1 General laws on consumer protection

In general, administrative, civil and criminal penalties may apply if providers of stem cells do not comply with legal requirements concerning the advertisement and promotion of products or services in China (Table 1). General laws such as the Protection of Consumer Rights and Interests Law (PCRIL)^17^ and Chinese Advertising Law (CAL)^18^ apply to the advertising of any goods or services and protect the interests of consumers. The PCRIL explicitly stipulates that if business operators present commodities or services through false advertising or any other means of misleading promotion, consumers may request the competent administrative departments to punish the advertising agents or publishers who engaged in false advertising.^19^ The CAL stipulates that advertising content shall be expressed in a true and lawful manner^20^ and that advertisements shall not have any false or misleading content to defraud or mislead consumers.^21^

**Table 1:** Existing laws and regulations that can be put into use to regulate advertising and promotion of stem cell–based interventions in China

Regulatory area	Key laws and regulations	Enforcement agencies
Regulating advertising activities and protecting consumers in general	The Protection of Consumer Rights and Interests Law; Chinese Advertising Law	State Administration for Market Regulation^a^
Regulating advertising and promotion of medical products	Chinese Advertising Law; Pharmaceutical Administration Law; Interim Measures for the Administration of Internet Advertising	National Medical Products Administration^b^ State Administration for Market Regulation
Regulating advertising and promotion of medical services	Chinese Advertising Law; Measures for the Administration of Medical Advertisements; Interim Measures for the Administration of Internet Advertising	National Health Commission^c^ State Administration for Market Regulation

Abbreviations: NHC, National Health Commission; NMPA, National Medical Products Administration; SAMR, State Administration for Market Regulation.

^a^ SAMR holds the power to review advertising activities in general and penalize those involved in false advertising, including those take part in false advertising of drug and medical services.

^b^ NMPA holds the power to review drug advertisements and supervise pharmaceutical companies.

^c^ NHC holds the power to review medical advertisements and supervise medical institutions.

Regarding false or misleading advertisements, the CAL defines a false advertisement as any advertisement that defrauds or misleads consumers through any false or misleading content.^22^ It also enumerates a list of false advertisement categories. Specifically, it is false advertising when any of the following occurs: (a) the advertised good or service does not exist; (b) information provided in the advertisement, such as a good's performance, functions, place of production, uses, quality, specification, ingredients, price, producer, term of validity, sales condition and honours received, as well as any commitments made concerning the good or service, is inconsistent with the actual circumstances and has substantial impact on purchases; (c) any scientific research result, statistical data, investigation result, excerpt, quotation or other information which is fabricated or forged or cannot be validated, yet has been used as a certification material; (d) the results of using the good or receiving the service are fabricated; and (e) consumers are otherwise defrauded or misled with any false or misleading content.^23^ These provisions, in principle, restrict advertising and promotionto prevent them being false or misleading, particularly to protect consumers. On the basis of these provisions,the advertising and promotion of unproven stem cell–based interventions in China may be regarded as false advertising under the CAL.

In addition, to promote fair market competition and protect legitimate business operators, the Anti‐Unfair Competition Law (AUCL) prohibits false advertising by business operators.^24^ According to the AUCL, a business shall not conduct any false or misleading commercial publicity with respect to the performance, functionality, quality, sales, product reviews and honours received of its commodities to defraud or mislead consumers. Neither shall a business help another business conduct any false or misleading commercial publicity by organizing false transaction or through any other means.^25^

Furthermore, the Chinese Criminal Law (CCL)^26^ has a provision for the crime of false advertising. The CCL stipulates that where, in violation of the State regulations, an advertiser, advertising agent or advertisement publisher, who uses advertisements to publicize goods or services falsely, and when the circumstances are serious, he/she shall be sentenced to not more than 2 years of fixed‐term imprisonment or criminal detention, and may in addition or exclusively be fined.^27^

In what follows, we introduce and analyze more specific legal and regulatory measures that exist in China regulating, respectively, the advertising of pharmaceutical products and medical services.

#### 3.2.2 Regulations for the advertisement and promotion of pharmaceutical products

In addition to aforementioned general instructions to all advertisers, the CAL contains relatively specific provisions regarding the particularity of pharmaceutical advertisements. Article 16 stipulates that advertisements for medical services, drugs or medical instruments shall not contain (a) any assertion or assurance regarding efficacy or safety; (b) any statement regarding the recovery or response rate; (c) any comparison with other drugs or medical instruments with respect to efficacy and safety, or any comparison with other medical institutions; (d) any recommendation or verification by an endorser; or (e) any other information prohibited by any lawor administrative regulation.^28^ Moreover, Article 40 of the CAL prohibits medical and pharmaceutical advertisements targeting minors.

Other laws, including the Pharmaceutical Administration Law (PAL), Regulations for the Implementation of the PAL and Measures for the Classification Management of Prescription Drugs and Over‐the‐counter Drugs (Trial) (hereafter, MCM‐PDOD),^29^ may also be applied to the advertising and promotion of pharmaceutical products. For instance, the PAL specifies that pharmaceutical advertisements shall not contain any assertion or guarantee regarding the effects or safety and that products/services shall not be endorsed or attested to by using the names or images of government bodies, research institutions, academic organizations, industry associations, experts, scholars, physicians, pharmacists or patients.^30^

The MCM‐PDOD also stipulates that prescription drugs can only be advertised in the professional medical press.^31^ Thus, in China, DTCA for prescription drugs is prohibited. However, it remains unclear whether stem cell–based interventions are prescription drugs. If stem cell–based interventions are defined by law as a drug, the aforementioned laws and regulations ‐ CAL, PCRIL, AUCL and CCL ‐ are in place and be put into use to regulate advertising and promotion of those produdcts.

#### 3.2.3 Regulations for the advertisement and promotion of medical services

In addition to the five prohibitions on the content of medical advertising in the CAL mentioned above, several specific departmental regulations ‐ such as the Measures for the Administration of Medical Advertisements (MAMA)^32^ and Interim Measures for the Administration of Internet Advertising^33^ ‐ also contain clauses that regulate the advertisement and promotion of medical services. The MAMA stipulates that no non‐medical institutionmay release medical advertisements and that a medical institution shall not release medical advertisements in the name of its internal department or office.^34^

The MAMA also explicitly stipulates that the content of medical advertisements is limited to eight items, such as the first name and address of the medical institution.^35^ Crucially, eight types of medical advertisements are prohibited by the MAMA. Among these, the first three are the most relevant to stem cell–based interventions: (a) [advertisements] involving medical technologies, diagnostic methods, names of diseases or drugs; (b) [advertisements] guaranteeing a cure or guaranteeing a cure in a concealed form; and (c) [advertisements] propagandizing the cure rate, efficiency or other clinical results.^36^ In addition, Article 16 of the MAMA prohibits the use of news or special programmes (columns) covering medical information services to release medical advertisements or doing so in a disguised form.

Lastly, the Administrative Measures for Stem Cell Clinical Research (Trial) specifically prohibits medical institutions from advertising their stem cell clinical research or doing so in disguise.^37^

In summary, China does not have laws, regulations or guidelines specifically concerning the advertisement and promotion of stem cell–based interventions. Nevertheless, under the general legal framework for consumer protection relevant legal provisions do exist, yet are scattered among various laws, regulations and departmental rules. To effectively deploy these legal and regulatory provisions to combat false and misleading advertisement and promotion of unproven stem cell–based interventions, the legal status of stem cell–based interventions needs to be first clarified. We will discuss this and additional recommendations in the following.

## 4 PROBLEMS AND RECOMMENDATIONS

Our survey of China's legal and regulatory system suggests that if used effectively, the aforementioned legal and regulatory documents and administrative measures can curtail the direct‐to‐consumer advertising of unproven, unauthorized stem cell–based interventions in China. Nevertheless, our search of the Internet reveals the laws have not be wielded effectively and prompts us to identify the main factors contributing to the problem. Based on these analyses, we provide recommendations. Key factors, we find, are those related to the particularity of stem cell–based interventions and to the existing legal (and regulatory) documents and regulatory practices.

### 4.1 Particularity of stem cell–based interventions and the need to clarify its legal status and cover the research phase

As mentioned, in China, regulators of stem cell clinical research have made it increasingly clear that stem cell–based interventions are, and are regulated as, a ‘drug’; however, no legal opinion or court decision has yet specified the legal status of stem cell–based interventions in clinical use. The absence of definite legal status allows some to insist that a stem cell–based product is a medical technology, but this leaves it an open question under which existing law or regulation should a particular advertising activity involved in selling stem cell–based interventions be scrutinized in China today. Moreover, which regulatory agency or agencies bear primary responsibility for regulating advertising activities in this area is not clear.

Notably, experiences in countries such as the United States and Canada have shown that the problem of defining stem cell–based treatment's status is commonly shared among regulators around the world. The problem arises from the particularity of stem cell–based interventions whose ‘liveness’ distinguishes them from traditional drugs; thus, legal clarification is needed before regulating the market.^38^ Given that regulators, researchers and biopharma companies in China have increasingly reached a consensus that stem cell–based intervention should be regulated as a drug, we recommend specifically adopting this stance in relevant laws and regulations. Accordingly, the PAL would be able to effectively regulate the advertising and promotion of stem cell–based interventions in China.

Clarifying the legal status of stem cell–based interventions does not by default eliminate false and misleading advertisements and promotions of unproven, unauthorized stem cell–based interventions, especially during their research and development period. We thus recommend that the prohibition of advertising stem cell–based interventions—a clause that is included in the AMSCCR—should be expanded to all research, development and marketing activities in this field until such products gain market authorization from the National Medical Products Administration (NMPA) in China.

### 4.2 Specifying the legal definition of false advertising and its criteria

In surveying relevant legal and regulatory documents, we found that the legal concept of false advertising is too generic in Chinese laws and regulations and that the criteria for what constitutes false advertising are too vague or abstract to aid legal or regulatory practices. Given the particularities of stem cell–based interventions, specific criteria need to be developed to clarify what makes an advertisement or promotion unlawful in this area.

Regulators in China may learn from the experience of Australia, where specific regulations, namely the Australian advertising guidance for businesses involved with stem cells and other human cells or tissue products, have been developed to curtail false and misleading advertisements of stem cell and related products.^39^ Chinese regulators seeking to develop false advertising criteria should be careful to clarify what is meant by concepts such as advertised features, performance, misleadingness and deceptiveness. In addition, we recommend including criteria such as eliciting unrealistic expectations (hype) among target groups such as patients. For instance, if an advertisement or promotion misleads a certain percentage of its target population, it would be deemed false advertising.

In addition, as mentioned in the first section, educational materials are often repurposed as advertising materials. Whereas some educational materials are misused by marketers, others contain false information in the first place in an attempt to create sensational content that grabs the attention of online readers. We thus recommend that, at least in the case of stem cell–based interventions, a clause regarding ‘misleading marketing’ should be introduced in relevant regulations that recognize the damaging effect of presenting false or misleading information concerning stem cell research, products or markets.

### 4.3 Improving the effectiveness of existing laws and regulations by improving regulatory practices

A third area that needs to be improved to curtail the false and misleading advertisement and promotion of stem cell–based interventions is regulatory practice. Regulatory agencies in China have made considerable efforts to regulate stem cell clinical research, but the specific problem of the false and misleading advertisement and promotion of stem cell–based interventions has not yet received much regulatory attention. In 2016, the death of a cancer patient named Wei Zexi prompted the regulatory agencies to investigate the online advertising of unauthorized immunotherapies.^40^ Nevertheless, such investigations have not be routinized; thus, no major deterrent effect on false and misleading advertisements has been realized. We thus recommend that the relevant regulatory agencies include overseeing and regularly inspecting advertisements, especially those disseminated on the Internet and targeting particular groups such as patients, in their regulatory framework.

Given that a clear legal status for stem cell–based interventions has yet to be developed in China, for the time being, we recommend that regulatory activity be taken jointly by the NHC, State Administration for Market Regulation (SAMR) and NMPA. Other regulatory agencies may also be called upon in particular cases—for instance, when a false advertisement causes serious harm to a patient, the police and the court may intervene.

In addition, legal liability for violating existing laws and regulations are disproportionately small compared with the profit that may be earned from selling unproven, unauthorized stem cell–based interventions through false or misleading advertisements. For instance, according to Article 55 of the CAL, ‘Publishing false advertisements in violation of the provisions of this law… [will result in] a fine of up to five times […] the advertising costs […] or up to 1 million yuan (approximately US$150,000) if the advertising costs are obviously low’. By contrast, in the United States, Google was investigated for hosting false medical advertisements on its advertising system AdWords and was fined US$500 million.^41^ We propose increasing the penalties for false medical or pharmaceutical advertisements to the equivalent amount in China.

However, above all, what will make a substantial difference in regulating advertising and promotion activities in this area is for regulators to change the mode of regulation by shifting from a post‐event investigatory scheme to a system in which they take a more proactive role in preventing detrimental events from happening. For example, in the case of Wei Zexi, regulators thoroughly investigated the wrongdoers, including the search engine Baidu that misled Wei and his family into believing in the effectiveness of the advertised ‘immunotherapy’. However, by the time of the investigation, Wei and his family—and other patients and their families—had already been harmed by such false advertisements. In Wei's case, the causality between the advertisement and the induced harm was easy to establish. In other cases, patients or patient families may experience considerable difficulty in demonstrating that their harm is attributable to false or misleading advertising activities when considering, in particular, the complexity of stem cell–based interventions and the variability of patients' conditions. Instead of waiting for cases to be brought to court, regulatory agencies in China should take a more proactive role in keeping the market actors in check, thereby introducing proper law and order into this burgeoning market. A good example is seen in the United States Food and Drug Administration commencing legal action against Regenerative Medical Group, Inc., the Telehealth Medical Group Inc., and Jarald Henderson D.O. for ‘deceptive acts or practices and the making of false advertisements, in or affecting commerce’.^42^ Considering that China has established a social credit system that enables identifying, recording and tracking wrongdoers in all aspects of their socio‐economic lives,^43^ relevant regulators should consider establishing a ‘blacklist’ system to regulate false and misleading Internet‐based, direct‐to‐consumer advertising activities in this field.

## 5 CONCLUSION

While the world continues fighting the COVID‐19 pandemic and its associated infodemic, here, we took a deep dive into the phenomenon of false and misleading DTCA for novel therapies that have been a troublingly persistent blight on the field of stem cell research. Our analysis of the regulatory situation in China revealed that to effectively curtail medical misinformation that targets particular patient groups or the public in general, having relevant laws and regulations in place may be a good start but is not enough. The specificity of novel therapies and their complexities need to be considered when designing more effective regulations and intervention measures. Coordination among multiple regulatory agencies is often required, and a proactive regulatory mode, as opposed to a mode in which only post‐event investigations are conducted, is recommended to prevent misleading information from harming patients or the public interest. Given that Internet‐based, direct‐to‐consumer advertising of unproven, unauthorized stem cell–based interventions has a global reach and contributes to the phenomenon of ‘stem cell tourism’, we recommend further investigations into regulatory situations in other jurisdictions whose joint results will provide an evidentiary basis for a coordinated global regulatory effort to fight this particular infodemic.

## CONFLICT OF INTEREST

The authors declare no competing interests.

## AUTHOR CONTRIBUTIONS

J.‐W.L and Y.‐Y.S. contributed equally to manuscript design, data analysis, writing and finalizing. L.‐Q.S. contributed to manuscript revision. X.G. contributed to data collection and analysis. Y.‐J.P. contributed to manuscript design, writing, revision and finalizing.

## Data Availability

Data sharing is not applicable to this article as no new data were created or analysed in this study.
